# Supplementary material for: Expression of PD-1 and CTLA-4 Are Negative Prognostic Markers in Renal Cell Carcinoma
Source: J Clin Med. 2019 May 24;8(5):743. doi: 10.3390/jcm8050743 (PMC6572544; doi:10.3390/jcm8050743)
Supplement: Supplementary file 1 [file jcm-08-00743-s001.zip › jcm-502061 supplementary 2-Data.pdf]

RCC for Publication.sav

	Diagnosedatum_ Primär	Met_Earlies tDatum	Last_contact_ Datum	AgeAtDiagnosis	Diagnosis_to _lastcontact
1	24.06.2010	.	01.06.2012	55	23
2	18.08.2011	.	09.01.2012	67	4
3	17.10.2003	.	01.07.2012	73	104
4	15.02.2007	.	01.08.2012	81	65
5	20.07.2006	.	01.07.2012	47	71
6	04.05.2005	.	09.08.2005	69	3
7	05.03.2010	05.03.2010	16.10.2010	79	7
8	24.07.1998	12.07.2003	12.07.2003	62	59
9	14.02.2002	.	14.06.2010	80	100
10	09.10.2002	.	01.07.2012	42	116
11	24.10.2003	.	01.01.2007	51	38
12	27.11.2003	.	01.07.2012	58	103
13	11.06.2004	.	01.01.2007	69	30
14	09.06.2005	26.09.2008	01.07.2012	55	84
15	21.11.2005	.	01.07.2012	41	79
16	10.04.2006	.	29.05.2012	57	73
17	08.06.2006	.	01.07.2012	50	72
18	30.11.2007	.	01.08.2012	42	56
19	15.02.2010	.	10.02.2012	64	23
20	01.07.2010	.	01.07.2012	66	24
21	11.08.2010	.	31.07.2012	63	23
22	09.09.2010	.	11.01.2012	73	16
23	15.10.2010	.	23.03.2012	71	17
24	11.01.2011	.	09.03.2012	79	13
25	01.02.2011	.	23.05.2012	88	15
26	11.04.2011	.	01.07.2012	70	14
27	24.05.2011	.	01.07.2012	74	13
28	22.08.2011	.	01.08.2012	67	11
29	12.09.2011	.	01.07.2012	56	9
30	26.09.2005	.	01.08.2012	47	82
31	23.10.2006	.	01.07.2012	50	68
32	06.04.2011	.	09.05.2012	53	13
33	15.01.2005	.	19.11.2009	61	58
34	22.08.2008	.	01.08.2012	71	47
35	10.09.2002	08.01.2004	21.08.2004	72	23
36	15.05.2004	.	28.02.2012	75	93
37	18.05.2004	.	01.07.2012	62	97

	Survival_OS	Survival_DSS	Geschlecht	pT_grouped
1	,00	,00	1,00	1,00
2	,00	,00	2,00	1,00
3	,00	,00	1,00	1,00
4	,00	,00	1,00	1,00
5	,00	,00	2,00	1,00
6	1,00	,00	2,00	3,00
7	1,00	,00	2,00	3,00
8	1,00	1,00	2,00	1,00
9	1,00	,00	1,00	1,00
10	,00	,00	2,00	2,00
11	,00	,00	2,00	1,00
12	,00	,00	2,00	1,00
13	,00	,00	1,00	2,00
14	,00	,00	1,00	1,00
15	,00	,00	1,00	2,00
16	,00	,00	1,00	1,00
17	,00	,00	1,00	1,00
18	,00	,00	2,00	1,00
19	,00	,00	1,00	1,00
20	,00	,00	1,00	1,00
21	,00	,00	1,00	1,00
22	1,00	,00	2,00	3,00
23	,00	,00	2,00	3,00
24	1,00	,00	2,00	1,00
25	,00	,00	2,00	1,00
26	,00	,00	1,00	1,00
27	,00	,00	1,00	2,00
28	,00	,00	2,00	3,00
29	,00	,00	2,00	1,00
30	,00	,00	2,00	1,00
31	,00	,00	2,00	3,00
32	,00	,00	2,00	1,00
33	1,00	1,00	2,00	.
34	,00	,00	2,00	1,00
35	1,00	1,00	2,00	3,00
36	,00	,00	2,00	1,00
37	,00	,00	2,00	1,00

RCC for Publication.sav

	Morphology_coded	Grading_Paktuell April2013	ECOG	Prop_PD1
1	4,00	.	0	,00
2	4,00	3	0	,00
3	4,00	3	.	,00
4	4,00	2	1	,00
5	4,00	2	0	,00
6	4,00	3	0	,00
7	4,00	3	1	1,00
8	3,00	2	.	,00
9	3,00	2	.	,00
10	3,00	2	.	,00
11	3,00	2	.	,00
12	3,00	2	.	,00
13	3,00	2	0	,00
14	3,00	2	0	,00
15	3,00	3	0	,00
16	3,00	2	0	,00
17	3,00	2	1	,00
18	3,00	2	0	,00
19	3,00	2	1	,00
20	3,00	2	1	,00
21	3,00	2	0	,00
22	3,00	2	0	,00
23	3,00	3	0	,00
24	3,00	2	1	,00
25	3,00	2	0	,00
26	3,00	2	0	,00
27	3,00	2	0	,00
28	3,00	2	0	,00
29	3,00	2	.	,00
30	3,00	2	0	,00
31	3,00	2	0	,00
32	2,00	2	0	.
33	2,00	3	0	.
34	2,00	1	0	,00
35	2,00	2	0	,00
36	2,00	2	1	,00
37	2,00	2	0	,00

	Intens_PD1	PD1IRS	Prop_PDL1_TU	Intens_PDL1_TU	PDL1_TU_IRS
1	.	,00	,00	.	,00
2	.	,00	,00	.	,00
3	.	,00	2,00	1,00	2,00
4	.	,00	1,00	1,00	1,00
5	.	,00	,00	.	,00
6	.	,00	2,00	1,00	2,00
7	1,00	1,00	,00	.	,00
8	.	,00	,00	.	,00
9	.	,00	,00	.	,00
10	.	,00	,00	.	,00
11	.	,00	,00	.	,00
12	.	,00	,00	.	,00
13	.	,00	,00	.	,00
14	.	,00	,00	.	,00
15	.	,00	,00	.	,00
16	.	,00	,00	.	,00
17	.	,00	,00	.	,00
18	.	,00	,00	.	,00
19	.	,00	,00	.	,00
20	.	,00	,00	.	,00
21	.	,00	,00	.	,00
22	.	,00	,00	.	,00
23	.	,00	,00	.	,00
24	.	,00	,00	.	,00
25	.	,00	,00	.	,00
26	.	,00	,00	.	,00
27	.	,00	,00	.	,00
28	.	,00	,00	.	,00
29	.	,00	,00	.	,00
30	.	,00	2,00	1,00	2,00
31	.	,00	,00	.	,00
32	.	.	,00	.	,00
33	.	.	3,00	3,00	9,00
34	.	,00	.	.	.
35	.	,00	.	.	.
36	.	,00	,00	.	,00
37	.	,00	,00	.	,00

RCC for Publication.sav

	Prop_PDL1_Lymph	Intens_PDL1_Lymph	PDL1_Lymph_IRS	andreasProp_Lymph_CTLA4_TU
1	.	.	,00	,00
2	.	.	,00	,00
3	.	.	,00	1,00
4	.	.	,00	,00
5	2,00	1,00	1,00	10,00
6	2,00	2,00	4,00	10,00
7	.	.	,00	2,00
8	.	.	,00	,00
9	.	.	,00	,00
10	.	.	,00	,00
11	.	.	,00	,00
12	.	.	,00	,00
13	.	.	,00	,00
14	.	.	,00	,00
15	.	.	,00	,00
16	.	.	,00	,00
17	.	.	,00	,00
18	.	.	,00	,00
19	.	.	,00	,00
20	.	.	,00	,00
21	.	.	,00	,00
22	.	.	,00	,00
23	.	.	,00	,00
24	.	.	,00	,00
25	.	.	,00	,00
26	.	.	,00	,00
27	.	.	,00	,00
28	.	.	,00	,00
29	.	.	,00	,00
30	.	.	,00	,00
31	1,00	1,00	1,00	,00
32	.	.	,00	.
33	.	.	,00	,00
34	.	.	.	,00
35	.	.	.	.
36	.	.	,00	,00
37	.	.	,00	,00

	CD3medianbis2k5unddarüber
1	,00
2	,00
3	,00
4	,00
5	1,00
6	,00
7	,00
8	,00
9	,00
10	,00
11	,00
12	,00
13	,00
14	,00
15	,00
16	,00
17	,00
18	,00
19	,00
20	.
21	,00
22	,00
23	,00
24	,00
25	,00
26	,00
27	,00
28	,00
29	,00
30	,00
31	,00
32	,00
33	.
34	,00
35	,00
36	,00
37	,00

RCC for Publication.sav

	Diagnosedatum_Primär	Met_EarliestDatum	Last_contact_Datum	AgeAtDiagnosis	Diagnosis_to_lastcontact
38	28.09.2005	.	02.05.2012	71	79
39	06.10.2005	.	01.07.2012	58	80
40	31.10.2006	.	01.07.2012	64	68
41	15.02.2007	.	01.08.2012	58	65
42	18.04.2007	10.08.2011	01.07.2012	65	62
43	14.12.2007	.	01.07.2012	61	54
44	30.06.2008	.	24.04.2009	71	9
45	17.07.2008	.	17.12.2011	50	41
46	19.12.2008	.	01.07.2012	67	42
47	15.01.2009	.	01.07.2012	59	41
48	15.02.2009	.	01.07.2012	58	40
49	30.10.2009	28.07.2010	20.11.2010	92	12
50	02.11.2009	.	01.08.2012	57	32
51	15.01.2010	15.01.2010	17.11.2010	73	10
52	24.02.2010	29.09.2011	25.01.2012	61	23
53	27.04.2010	.	19.04.2012	64	23
54	09.06.2010	.	16.12.2010	68	6
55	15.08.2010	.	06.06.2012	56	21
56	19.04.2011	.	21.02.2012	53	10
57	30.06.2010	.	18.04.2012	72	21
58	07.02.2005	.	22.02.2012	71	84
59	30.06.2006	.	24.06.2007	78	11
60	10.01.2007	.	01.08.2012	56	66
61	15.10.2008	.	21.11.2008	73	1
62	02.03.2010	.	01.07.2012	76	27
63	04.11.2011	.	05.07.2012	77	8
64	08.12.2011	.	27.06.2012	70	6
65	15.07.2010	.	02.04.2012	60	20
66	21.02.2007	.	10.08.2011	80	53
67	04.06.2007	.	16.04.2012	74	58
68	01.07.2006	01.07.2006	25.02.2007	47	7
69	02.11.2006	.	23.10.2010	71	47
70	05.02.2009	.	01.07.2012	48	40
71	15.04.2010	.	21.05.2012	62	25
72	20.04.2006	.	24.04.2012	68	72
73	08.04.2003	.	30.06.2010	73	86
74	27.03.2007	.	01.07.2012	63	63

	Survival_OS	Survival_DSS	Geschlecht	pT_grouped
38	,00	,00	1,00	1,00
39	,00	,00	2,00	.
40	,00	,00	2,00	1,00
41	,00	,00	1,00	1,00
42	,00	,00	2,00	2,00
43	,00	,00	1,00	3,00
44	1,00	,00	2,00	1,00
45	,00	,00	2,00	1,00
46	,00	,00	2,00	1,00
47	,00	,00	1,00	1,00
48	,00	,00	2,00	1,00
49	1,00	,00	1,00	3,00
50	,00	,00	2,00	1,00
51	1,00	,00	2,00	.
52	,00	,00	2,00	1,00
53	,00	,00	2,00	1,00
54	1,00	,00	1,00	1,00
55	,00	,00	2,00	1,00
56	,00	,00	1,00	1,00
57	,00	,00	2,00	2,00
58	,00	,00	1,00	3,00
59	1,00	,00	1,00	1,00
60	,00	,00	2,00	1,00
61	1,00	,00	2,00	1,00
62	,00	,00	2,00	1,00
63	,00	,00	2,00	1,00
64	,00	,00	2,00	1,00
65	,00	,00	2,00	1,00
66	1,00	,00	2,00	2,00
67	,00	,00	1,00	1,00
68	1,00	1,00	2,00	3,00
69	1,00	,00	2,00	1,00
70	,00	,00	2,00	.
71	,00	,00	2,00	1,00
72	,00	,00	2,00	1,00
73	1,00	,00	2,00	1,00
74	,00	,00	1,00	.

	Morphology_coded	Grading_Paktuell April2013	ECOG	Prop_PD1
38	2,00	2	1	,00
39	2,00	2	0	,00
40	2,00	2	0	,00
41	2,00	2	0	,00
42	2,00	2	0	,00
43	2,00	2	2	,00
44	2,00	2	1	,00
45	2,00	2	0	,00
46	2,00	2	1	,00
47	2,00	1	0	,00
48	2,00	1	0	,00
49	2,00	3	0	,00
50	2,00	2	0	,00
51	2,00	2	0	,00
52	2,00	2	1	,00
53	2,00	2	1	,00
54	2,00	2	0	,00
55	2,00	2	0	,00
56	2,00	2	.	,00
57	2,00	2	0	,00
58	2,00	2	0	,00
59	2,00	2	1	,00
60	2,00	2	0	,00
61	2,00	2	1	,00
62	2,00	2	.	,00
63	2,00	2	0	,00
64	2,00	2	.	,00
65	2,00	2	1	,00
66	2,00	3	1	,00
67	2,00	3	0	,00
68	2,00	3	0	2,00
69	2,00	3	1	1,00
70	2,00	2	0	2,00
71	2,00	1	0	1,00
72	2,00	3	1	1,00
73	1,00	2	.	.
74	1,00	2	0	.

	Intens_PD1	PD1IRS	Prop_PDL1_TU	Intens_PDL1_TU	PDL1_TU_IRS
38	.	,00	,00	.	,00
39	.	,00	,00	.	,00
40	.	,00	,00	.	,00
41	.	,00	,00	.	,00
42	.	,00	,00	.	,00
43	.	,00	,00	.	,00
44	.	,00	,00	.	,00
45	.	,00	,00	.	,00
46	.	,00	,00	.	,00
47	.	,00	,00	.	,00
48	.	,00	,00	.	,00
49	.	,00	,00	.	,00
50	.	,00	,00	.	,00
51	.	,00	,00	.	,00
52	.	,00	,00	.	,00
53	.	,00	,00	.	,00
54	.	,00	,00	.	,00
55	.	,00	,00	.	,00
56	.	,00	,00	.	,00
57	.	,00	2,00	2,00	4,00
58	.	,00	2,00	1,00	2,00
59	.	,00	1,00	1,00	1,00
60	.	,00	2,00	2,00	4,00
61	.	,00	1,00	1,00	1,00
62	.	,00	2,00	1,00	2,00
63	.	,00	3,00	2,00	6,00
64	.	,00	2,00	2,00	4,00
65	.	,00	1,00	1,00	1,00
66	.	,00	,00	.	,00
67	.	,00	2,00	2,00	4,00
68	1,00	2,00	,00	.	,00
69	1,00	1,00	,00	.	,00
70	1,00	2,00	2,00	1,00	2,00
71	1,00	1,00	1,00	1,00	1,00
72	1,00	1,00	,00	.	,00
73	.	.	,00	.	,00
74	.	.	,00	.	,00

	Prop_PDL1_Lymph	Intens_PDL1_Lymph	PDL1_Lymph_IRS	andreasProp_Lymph_CTLA4_TU
38	.	.	,00	,00
39	.	.	,00	,00
40	.	.	,00	,00
41	.	.	,00	,00
42	.	.	,00	,00
43	.	.	,00	,00
44	.	.	,00	,00
45	.	.	,00	,00
46	.	.	,00	,00
47	.	.	,00	,00
48	.	.	,00	,00
49	.	.	,00	,00
50	.	.	,00	,00
51	.	.	,00	,00
52	.	.	,00	,00
53	.	.	,00	,00
54	.	.	,00	,00
55	.	.	,00	,00
56	.	.	,00	,00
57	.	.	,00	.
58	.	.	,00	,00
59	.	.	,00	,00
60	.	.	,00	,00
61	.	.	?	?
62	.	.	?	?
63	.	.	?	?
64	.	.	?	?
65	.	.	?	?
66	1,00	1,00	?	?
67	2,00	2,00	?	?
68	.	.	?	?
69	.	.	?	?
70	.	.	?	?
71	.	.	?	?
72	1,00	1,00	?	?
73	.	.	?	.
74	.	.	,00	.

	CD3medianbis2k5unddarüber
38	,00
39	,00
40	,00
41	,00
42	1,00
43	,00
44	,00
45	1,00
46	1,00
47	1,00
48	1,00
49	,00
50	1,00
51	,00
52	,00
53	1,00
54	,00
55	,00
56	,00
57	,00
58	,00
59	1,00
60	1,00
61	,00
62	1,00
63	,00
64	,00
65	1,00
66	1,00
67	,00
68	1,00
69	,00
70	,00
71	1,00
72	1,00
73	.
74	1,00

	Diagnosedatum_Primär	Met_EarliestDatum	Last_contact_Datum	AgeAtDiagnosis	Diagnosis_to_lastcontact
75	15.11.2007	.	01.07.2012	76	55
76	02.02.2009	.	01.07.2012	77	40
77	15.08.2010	.	21.05.2012	80	21
78	03.12.2001	.	13.04.2004	72	28
79	11.12.2001	.	27.03.2012	73	123
80	25.09.2006	.	07.02.2012	85	64
81	01.12.2009	.	01.07.2012	39	31
82	10.01.2005	.	24.02.2010	71	61
83	31.03.2010	.	18.06.2012	23	26
84	15.08.2010	.	30.03.2012	55	19
85	12.01.2011	.	02.03.2011	60	1
86	26.11.2004	.	15.02.2012	65	86
87	02.02.2009	02.02.2009	09.09.2009	66	7
88	30.11.2011	30.11.2011	05.01.2012	73	1
89	26.07.2004	.	01.07.2012	63	95
90	28.12.2005	.	01.07.2012	43	78
91	01.02.2008	01.02.2008	11.04.2009	69	14
92	15.12.2009	.	23.05.2012	74	29
93	15.12.2001	.	01.07.2012	64	126
94	19.12.2001	30.06.2004	28.09.2004	85	33
95	18.01.2002	.	01.07.2012	54	125
96	19.01.2002	.	01.07.2012	72	125
97	04.02.2002	.	01.08.2012	60	125
98	19.03.2002	.	03.04.2004	78	24
99	22.04.2002	.	01.07.2012	56	122
100	15.05.2002	15.05.2002	24.07.2003	70	14
101	21.06.2002	17.12.2002	27.05.2003	64	11
102	08.07.2002	.	01.07.2012	48	119
103	16.01.2003	.	04.11.2011	50	105
104	09.04.2003	09.04.2003	01.07.2012	60	110
105	11.04.2003	26.06.2004	24.10.2008	65	66
106	11.04.2003	26.06.2004	24.10.2008	65	66
107	27.05.2003	.	25.06.2009	77	72
108	16.06.2003	.	01.07.2012	56	108
109	26.06.2003	.	01.07.2012	75	108
110	30.06.2003	.	01.07.2012	62	108
111	09.07.2003	.	01.01.2007	79	41

	Survival_OS	Survival_DSS	Geschlecht	pT_grouped
75	,00	,00	1,00	1,00
76	,00	,00	2,00	1,00
77	,00	,00	1,00	1,00
78	1,00	1,00	2,00	1,00
79	,00	,00	2,00	1,00
80	,00	,00	2,00	1,00
81	,00	,00	2,00	1,00
82	1,00	,00	2,00	1,00
83	,00	,00	2,00	1,00
84	,00	,00	1,00	1,00
85	1,00	,00	2,00	1,00
86	,00	,00	2,00	1,00
87	1,00	1,00	1,00	2,00
88	,00	,00	2,00	1,00
89	,00	,00	1,00	1,00
90	,00	,00	2,00	3,00
91	1,00	1,00	2,00	1,00
92	,00	,00	1,00	3,00
93	,00	,00	1,00	1,00
94	1,00	1,00	1,00	2,00
95	,00	,00	2,00	1,00
96	,00	,00	2,00	2,00
97	,00	,00	2,00	1,00
98	1,00	1,00	1,00	3,00
99	,00	,00	2,00	1,00
100	1,00	,00	2,00	1,00
101	1,00	1,00	2,00	2,00
102	,00	,00	2,00	3,00
103	1,00	,00	2,00	1,00
104	,00	,00	2,00	3,00
105	1,00	,00	2,00	1,00
106	1,00	,00	2,00	3,00
107	1,00	1,00	1,00	1,00
108	,00	,00	2,00	1,00
109	,00	,00	2,00	1,00
110	,00	,00	1,00	1,00
111	,00	,00	1,00	3,00

	Morphology_coded	Grading_Paktuell April2013	ECOG	Prop_PD1
75	1,00	1	0	.
76	1,00	1	0	.
77	1,00	1	1	.
78	1,00	2	.	.
79	1,00	3	2	.
80	1,00	2	.	.
81	1,00	2	.	.
82	1,00	2	1	.
83	1,00	2	0	,00
84	1,00	1	.	,00
85	1,00	2	0	,00
86	1,00	1	0	,00
87	1,00	3	1	,00
88	1,00	2	0	,00
89	1,00	2	0	,00
90	1,00	2	0	,00
91	1,00	2	3	,00
92	1,00	2	0	,00
93	1,00	2	.	,00
94	1,00	3	0	,00
95	1,00	2	.	,00
96	1,00	2	.	,00
97	1,00	2	.	,00
98	1,00	2	.	,00
99	1,00	2	.	,00
100	1,00	2	.	,00
101	1,00	3	.	,00
102	1,00	2	.	,00
103	1,00	2	0	,00
104	1,00	2	.	,00
105	1,00	2	.	,00
106	1,00	2	.	,00
107	1,00	2	.	,00
108	1,00	3	.	,00
109	1,00	1	.	,00
110	1,00	2	.	,00
111	1,00	2	.	,00

	Intens_PD1	PD1IRS	Prop_PDL1_TU	Intens_PDL1_TU	PDL1_TU_IRS
75	.	.	,00	.	,00
76	.	.	,00	.	,00
77	.	.	,00	.	,00
78	.	.	,00	.	,00
79	.	.	,00	.	,00
80	.	.	,00	.	,00
81	.	.	,00	.	,00
82	.	.	,00	.	,00
83	.	,00	.	.	.
84	.	,00	.	.	.
85	.	,00	.	.	.
86	.	,00	.	.	.
87	.	,00	.	.	.
88	.	,00	.	.	.
89	.	,00	,00	.	,00
90	.	,00	,00	.	,00
91	.	,00	,00	.	,00
92	.	,00	,00	.	,00
93	.	,00	,00	.	,00
94	.	,00	,00	.	,00
95	.	,00	,00	.	,00
96	.	,00	,00	.	,00
97	.	,00	,00	.	,00
98	.	,00	,00	.	,00
99	.	,00	,00	.	,00
100	.	,00	,00	.	,00
101	.	,00	,00	.	,00
102	.	,00	,00	.	,00
103	.	,00	,00	.	,00
104	.	,00	,00	.	,00
105	.	,00	,00	.	,00
106	.	,00	,00	.	,00
107	.	,00	,00	.	,00
108	.	,00	,00	.	,00
109	.	,00	,00	.	,00
110	.	,00	,00	.	,00
111	.	,00	,00	.	,00

	Prop_PDL1_Lymph	Intens_PDL1_Lymph	PDL1_Lymph_IRS	andreasProp_Lymph_CTLA4_TU
75	.	.	,00	.
76	.	.	,00	.
77	.	.	,00	.
78	.	.	,00	,00
79	.	.	,00	,00
80	.	.	,00	,00
81	.	.	,00	,00
82	2,00	2,00	4,00	,00
83	.	.	.	.
84	.	.	.	.
85	.	.	.	.
86	.	.	.	,00
87	.	.	.	,00
88	.	.	.	10,00
89	.	.	,00	.
90	.	.	,00	.
91	.	.	,00	.
92	.	.	,00	.
93	.	.	,00	,00
94	.	.	,00	,00
95	.	.	,00	,00
96	.	.	,00	,00
97	.	.	,00	1,00
98	.	.	,00	,00
99	.	.	,00	,00
100	.	.	,00	,00
101	.	.	,00	,00
102	.	.	,00	,00
103	.	.	,00	,00
104	.	.	,00	,00
105	.	.	,00	,00
106	.	.	,00	,00
107	.	.	,00	,00
108	.	.	,00	,00
109	.	.	,00	,00
110	.	.	,00	,00
111	.	.	,00	,00

	CD3medianbis2k5unddarüber
75	1,00
76	,00
77	.
78	1,00
79	1,00
80	1,00
81	1,00
82	1,00
83	.
84	,00
85	,00
86	,00
87	,00
88	,00
89	1,00
90	1,00
91	1,00
92	1,00
93	,00
94	,00
95	1,00
96	,00
97	1,00
98	1,00
99	,00
100	,00
101	,00
102	1,00
103	.
104	,00
105	1,00
106	,00
107	1,00
108	1,00
109	1,00
110	1,00
111	,00

	Diagnosedatum_Primär	Met_EarliestDatum	Last_contact_Datum	AgeAtDiagnosis	Diagnosis_to_lastcontact
112	20.10.2003	.	01.07.2012	68	104
113	28.10.2003	.	01.01.2007	66	38
114	28.10.2003	.	01.07.2012	45	104
115	29.10.2003	.	01.08.2012	57	105
116	15.11.2003	08.04.2008	01.08.2009	51	68
117	19.11.2003	.	01.07.2012	58	103
118	27.11.2003	10.08.2009	30.03.2011	56	88
119	16.12.2003	.	20.07.2007	76	43
120	17.12.2003	.	01.07.2012	72	102
121	14.01.2004	.	23.05.2012	60	100
122	12.05.2004	12.05.2004	06.02.2008	78	44
123	14.05.2004	.	04.11.2008	54	53
124	19.05.2004	.	19.08.2008	64	51
125	26.05.2004	19.02.2009	12.07.2012	63	97
126	27.05.2004	27.05.2004	07.01.2011	62	79
127	03.06.2004	.	01.07.2012	66	96
128	17.06.2004	.	01.07.2012	67	96
129	21.06.2004	13.10.2005	04.07.2007	66	36
130	15.07.2004	22.10.2004	09.03.2005	72	7
131	15.07.2004	.	12.01.2008	40	41
132	26.11.2004	28.04.2008	24.02.2012	75	86
133	07.12.2004	07.12.2004	14.06.2006	66	18
134	13.12.2004	13.12.2004	01.07.2012	70	90
135	15.12.2004	.	15.02.2012	73	86
136	14.01.2005	.	01.07.2012	67	89
137	02.02.2005	02.02.2005	01.07.2012	44	88
138	03.02.2005	.	06.11.2007	67	33
139	18.02.2005	.	15.02.2012	44	83
140	14.04.2005	.	01.07.2012	65	86
141	11.05.2005	11.05.2005	26.10.2008	68	41
142	03.06.2005	.	22.07.2010	63	61
143	08.06.2005	.	07.07.2011	64	72
144	01.07.2005	.	01.07.2012	48	84
145	05.07.2005	.	01.08.2012	64	84
146	11.07.2005	11.07.2005	30.12.2005	68	5
147	19.07.2005	.	06.03.2012	52	79
148	22.08.2005	.	01.08.2012	58	83

	Survival_OS	Survival_DSS	Geschlecht	pT_grouped
112	,00	,00	2,00	.
113	,00	,00	1,00	.
114	,00	,00	1,00	.
115	,00	,00	2,00	.
116	1,00	1,00	2,00	2,00
117	,00	,00	2,00	1,00
118	1,00	,00	2,00	3,00
119	1,00	,00	2,00	3,00
120	,00	,00	2,00	1,00
121	,00	,00	1,00	1,00
122	1,00	1,00	2,00	3,00
123	1,00	1,00	2,00	1,00
124	1,00	,00	2,00	1,00
125	,00	,00	2,00	1,00
126	1,00	,00	2,00	2,00
127	,00	,00	1,00	1,00
128	,00	,00	1,00	1,00
129	1,00	1,00	2,00	1,00
130	1,00	,00	1,00	3,00
131	1,00	,00	2,00	3,00
132	1,00	,00	2,00	1,00
133	1,00	1,00	2,00	2,00
134	,00	,00	1,00	3,00
135	,00	,00	2,00	1,00
136	,00	,00	1,00	1,00
137	,00	,00	2,00	3,00
138	1,00	,00	1,00	3,00
139	,00	,00	2,00	1,00
140	,00	,00	1,00	3,00
141	1,00	1,00	1,00	1,00
142	1,00	,00	2,00	3,00
143	,00	,00	1,00	1,00
144	,00	,00	1,00	1,00
145	,00	,00	2,00	1,00
146	1,00	,00	1,00	3,00
147	,00	,00	2,00	1,00
148	,00	,00	1,00	1,00

	Morphology_coded	Grading_Paktuell April2013	ECOG	Prop_PD1
112	1,00	2	.	,00
113	1,00	2	.	,00
114	1,00	2	.	,00
115	1,00	2	.	,00
116	1,00	1	.	,00
117	1,00	2	.	,00
118	1,00	2	.	,00
119	1,00	3	1	,00
120	1,00	2	0	,00
121	1,00	2	0	,00
122	1,00	2	1	,00
123	1,00	2	1	,00
124	1,00	2	1	,00
125	1,00	3	0	,00
126	1,00	3	0	,00
127	1,00	2	0	,00
128	1,00	2	1	,00
129	1,00	2	1	,00
130	1,00	3	1	,00
131	1,00	2	0	,00
132	1,00	2	0	,00
133	1,00	3	0	,00
134	1,00	2	.	,00
135	1,00	2	1	,00
136	1,00	2	0	,00
137	1,00	3	0	,00
138	1,00	2	0	,00
139	1,00	1	0	,00
140	1,00	2	0	,00
141	1,00	2	0	,00
142	1,00	2	1	,00
143	1,00	2	1	,00
144	1,00	1	0	,00
145	1,00	2	1	,00
146	1,00	3	1	,00
147	1,00	2	1	,00
148	1,00	2	1	,00

	Intens_PD1	PD1IRS	Prop_PDL1_TU	Intens_PDL1_TU	PDL1_TU_IRS
112	.	,00	,00	.	,00
113	.	,00	,00	.	,00
114	.	,00	,00	.	,00
115	.	,00	,00	.	,00
116	.	,00	,00	.	,00
117	.	,00	,00	.	,00
118	.	,00	,00	.	,00
119	.	,00	,00	.	,00
120	.	,00	,00	.	,00
121	.	,00	,00	.	,00
122	.	,00	,00	.	,00
123	.	,00	,00	.	,00
124	.	,00	,00	.	,00
125	.	,00	,00	.	,00
126	.	,00	,00	.	,00
127	.	,00	,00	.	,00
128	.	,00	,00	.	,00
129	.	,00	,00	.	,00
130	.	,00	,00	.	,00
131	.	,00	,00	.	,00
132	.	,00	,00	.	,00
133	.	,00	,00	.	,00
134	.	,00	,00	.	,00
135	.	,00	,00	.	,00
136	.	,00	,00	.	,00
137	.	,00	,00	.	,00
138	.	,00	,00	.	,00
139	.	,00	,00	.	,00
140	.	,00	,00	.	,00
141	.	,00	,00	.	,00
142	.	,00	,00	.	,00
143	.	,00	,00	.	,00
144	.	,00	,00	.	,00
145	.	,00	,00	.	,00
146	.	,00	,00	.	,00
147	.	,00	,00	.	,00
148	.	,00	,00	.	,00

RCC for Publication.sav

	Prop_PDL1_Lymph	Intens_PDL1_Lymph	PDL1_Lymph_IRS	andreasProp_Lymph_CTLA4_TU
112	.	.	,00	,00
113	.	.	,00	,00
114	.	.	,00	,00
115	.	.	,00	2,00
116	.	.	,00	,00
117	.	.	,00	,00
118	.	.	,00	,00
119	.	.	,00	,00
120	.	.	,00	,00
121	.	.	,00	,00
122	.	.	,00	,00
123	.	.	,00	,00
124	.	.	,00	,00
125	.	.	,00	,00
126	.	.	,00	,00
127	.	.	,00	,00
128	.	.	,00	,00
129	.	.	,00	,00
130	.	.	,00	,00
131	.	.	,00	,00
132	.	.	,00	,00
133	.	.	,00	,00
134	.	.	,00	,00
135	.	.	,00	,00
136	.	.	,00	,00
137	.	.	,00	,00
138	.	.	,00	,00
139	.	.	,00	,00
140	.	.	,00	,00
141	.	.	,00	,00
142	.	.	,00	,00
143	.	.	,00	,00
144	.	.	,00	,00
145	.	.	,00	,00
146	.	.	,00	,00
147	.	.	,00	,00
148	.	.	,00	,00

	CD3medianbis2k5unddarüber
112	,00
113	1,00
114	,00
115	1,00
116	1,00
117	1,00
118	,00
119	,00
120	,00
121	1,00
122	,00
123	,00
124	,00
125	1,00
126	1,00
127	1,00
128	,00
129	,00
130	,00
131	,00
132	1,00
133	1,00
134	1,00
135	1,00
136	1,00
137	1,00
138	,00
139	,00
140	,00
141	,00
142	,00
143	,00
144	1,00
145	1,00
146	,00
147	,00
148	,00

RCC for Publication.sav

	Diagnosedatum_Primär	Met_EarliestDatum	Last_contact_Datum	AgeAtDiagnosis	Diagnosis_to_lastcontact
149	23.08.2005	.	18.08.2006	65	11
150	08.09.2005	.	01.08.2012	63	82
151	08.09.2005	.	01.08.2012	72	82
152	11.10.2005	26.05.2008	16.02.2010	80	52
153	14.10.2005	.	06.06.2012	74	79
154	18.10.2005	.	01.07.2012	62	80
155	03.11.2005	.	01.07.2012	66	79
156	14.11.2005	.	10.05.2012	48	77
157	13.12.2005	09.04.2009	10.05.2012	65	76
158	28.12.2005	28.12.2005	12.03.2006	77	2
159	15.01.2006	.	22.03.2007	61	14
160	15.01.2006	.	01.07.2012	76	77
161	24.01.2006	15.11.2006	21.12.2006	67	10
162	27.01.2006	.	01.08.2012	66	78
163	06.02.2006	.	01.07.2012	56	76
164	21.03.2006	.	01.08.2012	79	76
165	10.04.2006	.	01.09.2011	65	64
166	18.04.2006	15.08.2007	19.04.2012	71	72
167	24.04.2006	24.04.2006	08.12.2006	69	7
168	26.04.2006	.	20.04.2009	53	35
169	02.05.2006	02.05.2006	22.04.2007	66	11
170	05.05.2006	.	11.06.2012	68	73
171	16.05.2006	.	01.07.2012	69	73
172	08.06.2006	.	01.07.2012	50	72
173	14.06.2006	.	12.05.2012	83	70
174	19.06.2006	.	08.05.2012	80	70
175	24.07.2006	.	01.07.2012	56	71
176	08.12.2006	19.06.2008	14.08.2009	54	32
177	15.12.2006	31.07.2008	16.11.2008	62	23
178	29.12.2006	.	01.07.2012	55	66
179	07.02.2007	.	08.05.2012	68	63
180	19.02.2007	.	01.08.2012	64	65
181	09.03.2007	.	01.07.2012	56	63
182	15.04.2007	.	01.08.2012	67	63
183	18.04.2007	10.08.2011	01.07.2012	65	62
184	03.05.2007	.	01.07.2012	64	61
185	09.05.2007	.	01.07.2012	66	61

	Survival_OS	Survival_DSS	Geschlecht	pT_grouped
149	1,00	,00	2,00	1,00
150	,00	,00	1,00	1,00
151	,00	,00	2,00	3,00
152	1,00	1,00	2,00	1,00
153	,00	,00	2,00	3,00
154	,00	,00	2,00	.
155	,00	,00	1,00	1,00
156	,00	,00	2,00	1,00
157	,00	,00	2,00	3,00
158	1,00	1,00	2,00	1,00
159	,00	,00	2,00	1,00
160	,00	,00	1,00	3,00
161	1,00	1,00	1,00	2,00
162	,00	,00	2,00	1,00
163	,00	,00	2,00	1,00
164	,00	,00	1,00	1,00
165	1,00	,00	2,00	1,00
166	,00	,00	2,00	2,00
167	1,00	1,00	2,00	3,00
168	1,00	,00	2,00	3,00
169	1,00	,00	2,00	3,00
170	,00	,00	2,00	1,00
171	,00	,00	2,00	1,00
172	,00	,00	2,00	3,00
173	,00	,00	1,00	3,00
174	,00	,00	2,00	1,00
175	,00	,00	2,00	1,00
176	1,00	1,00	2,00	2,00
177	1,00	1,00	1,00	3,00
178	,00	,00	1,00	2,00
179	,00	,00	1,00	1,00
180	,00	,00	1,00	1,00
181	,00	,00	2,00	1,00
182	,00	,00	1,00	1,00
183	,00	,00	2,00	3,00
184	,00	,00	1,00	1,00
185	,00	,00	2,00	3,00

	Morphology_coded	Grading_Paktuell April2013	ECOG	Prop_PD1
149	1,00	2	0	,00
150	1,00	2	0	,00
151	1,00	2	0	,00
152	1,00	3	0	,00
153	1,00	2	0	,00
154	1,00	3	0	,00
155	1,00	3	0	,00
156	1,00	1	0	,00
157	1,00	2	.	,00
158	1,00	3	0	,00
159	1,00	2	0	,00
160	1,00	2	0	,00
161	1,00	3	0	,00
162	1,00	3	0	,00
163	1,00	2	0	,00
164	1,00	2	0	,00
165	1,00	2	0	,00
166	1,00	3	0	,00
167	1,00	3	2	,00
168	1,00	3	1	,00
169	1,00	3	0	,00
170	1,00	2	1	,00
171	1,00	2	2	,00
172	1,00	2	0	,00
173	1,00	2	0	,00
174	1,00	1	0	,00
175	1,00	3	0	,00
176	1,00	3	0	,00
177	1,00	3	1	,00
178	1,00	2	0	,00
179	1,00	2	1	,00
180	1,00	2	0	,00
181	1,00	2	0	,00
182	1,00	1	0	,00
183	1,00	2	0	,00
184	1,00	2	0	,00
185	1,00	2	0	,00

	Intens_PD1	PD1IRS	Prop_PDL1_TU	Intens_PDL1_TU	PDL1_TU_IRS
149	.	,00	,00	.	,00
150	.	,00	,00	.	,00
151	.	,00	,00	.	,00
152	.	,00	,00	.	,00
153	.	,00	,00	.	,00
154	.	,00	,00	.	,00
155	.	,00	,00	.	,00
156	.	,00	,00	.	,00
157	.	,00	,00	.	,00
158	.	,00	,00	.	,00
159	.	,00	,00	.	,00
160	.	,00	,00	.	,00
161	.	,00	,00	.	,00
162	.	,00	,00	.	,00
163	.	,00	,00	.	,00
164	.	,00	,00	.	,00
165	.	,00	,00	.	,00
166	.	,00	,00	.	,00
167	.	,00	,00	.	,00
168	.	,00	,00	.	,00
169	.	,00	,00	.	,00
170	.	,00	,00	.	,00
171	.	,00	,00	.	,00
172	.	,00	,00	.	,00
173	.	,00	,00	.	,00
174	.	,00	,00	.	,00
175	.	,00	,00	.	,00
176	.	,00	,00	.	,00
177	.	,00	,00	.	,00
178	.	,00	,00	.	,00
179	.	,00	,00	.	,00
180	.	,00	,00	.	,00
181	.	,00	,00	.	,00
182	.	,00	,00	.	,00
183	.	,00	,00	.	,00
184	.	,00	,00	.	,00
185	.	,00	,00	.	,00

	Prop_PDL1_Lymph	Intens_PDL1_Lymph	PDL1_Lymph_IRS	andreasProp_Lymph_CTLA4_TU
149	.	.	,00	,00
150	.	.	,00	,00
151	.	.	,00	,00
152	.	.	,00	,00
153	.	.	,00	,00
154	.	.	,00	,00
155	.	.	,00	,00
156	.	.	,00	,00
157	.	.	,00	,00
158	.	.	,00	,00
159	.	.	,00	,00
160	.	.	,00	,00
161	.	.	,00	2,00
162	.	.	,00	,00
163	.	.	,00	,00
164	.	.	,00	1,00
165	.	.	,00	,00
166	.	.	,00	,00
167	.	.	,00	,00
168	.	.	,00	,00
169	.	.	,00	,00
170	.	.	,00	,00
171	.	.	,00	,00
172	.	.	,00	,00
173	.	.	,00	,00
174	.	.	,00	,00
175	.	.	,00	,00
176	.	.	,00	,00
177	.	.	,00	,00
178	.	.	,00	,00
179	.	.	,00	,00
180	.	.	,00	,00
181	.	.	,00	,00
182	.	.	,00	,00
183	.	.	,00	,00
184	.	.	,00	,00
185	.	.	,00	,00

	CD3medianbis2k5unddarüber
149	1,00
150	,00
151	,00
152	,00
153	,00
154	,00
155	,00
156	1,00
157	,00
158	1,00
159	.
160	1,00
161	,00
162	.
163	1,00
164	,00
165	1,00
166	1,00
167	1,00
168	1,00
169	1,00
170	1,00
171	1,00
172	,00
173	,00
174	1,00
175	,00
176	,00
177	1,00
178	1,00
179	,00
180	,00
181	,00
182	1,00
183	1,00
184	,00
185	1,00

	Diagnosedatum_Primär	Met_EarliestDatum	Last_contact_Datum	AgeAtDiagnosis	Diagnosis_to_lastcontact
186	15.05.2007	.	01.08.2012	79	62
187	09.06.2007	01.06.2009	01.06.2009	76	23
188	14.06.2007	.	02.03.2012	65	56
189	21.06.2007	.	01.07.2012	66	60
190	11.07.2007	.	01.07.2012	84	59
191	20.07.2007	19.02.2010	27.05.2011	61	46
192	25.08.2007	.	02.03.2009	68	18
193	05.09.2007	.	06.06.2012	81	57
194	11.09.2007	.	01.07.2012	59	57
195	14.09.2007	.	01.07.2012	74	57
196	27.09.2007	10.02.2008	01.07.2008	50	9
197	17.10.2007	17.10.2007	23.03.2008	76	5
198	15.11.2007	14.03.2008	28.06.2008	78	7
199	27.11.2007	.	01.07.2012	58	55
200	03.12.2007	.	12.04.2012	50	52
201	03.12.2007	.	09.09.2009	62	21
202	14.12.2007	.	01.07.2012	73	54
203	04.01.2008	29.05.2008	16.05.2012	56	52
204	23.01.2008	.	01.07.2012	70	53
205	03.02.2008	07.04.2010	19.06.2010	70	28
206	13.02.2008	.	01.07.2012	48	52
207	14.02.2008	.	06.06.2012	74	51
208	06.03.2008	06.03.2008	03.03.2009	45	11
209	12.03.2008	.	01.07.2012	65	51
210	14.03.2008	14.03.2008	30.04.2008	41	1
211	19.03.2008	.	01.02.2012	62	46
212	09.05.2008	.	04.07.2012	52	49
213	13.05.2008	09.09.2010	21.05.2012	47	48
214	27.05.2008	.	01.08.2012	73	50
215	03.06.2008	.	23.05.2012	67	47
216	23.07.2008	.	16.02.2012	75	42
217	11.08.2008	.	19.04.2009	76	8
218	26.08.2008	.	01.08.2012	73	47
219	27.08.2008	.	27.02.2012	73	42
220	18.09.2008	.	11.06.2009	73	8
221	08.10.2008	.	01.08.2012	83	45
222	10.10.2008	.	29.01.2009	70	3

	Survival_OS	Survival_DSS	Geschlecht	pT_grouped
186	,00	,00	1,00	1,00
187	1,00	,00	1,00	2,00
188	,00	,00	2,00	2,00
189	,00	,00	1,00	1,00
190	,00	,00	1,00	1,00
191	1,00	,00	2,00	3,00
192	1,00	,00	2,00	1,00
193	,00	,00	2,00	1,00
194	,00	,00	1,00	3,00
195	,00	,00	1,00	1,00
196	1,00	1,00	2,00	2,00
197	1,00	1,00	1,00	3,00
198	1,00	1,00	2,00	3,00
199	,00	,00	2,00	1,00
200	,00	,00	2,00	1,00
201	1,00	,00	2,00	3,00
202	,00	,00	1,00	1,00
203	,00	,00	1,00	2,00
204	,00	,00	1,00	1,00
205	1,00	,00	1,00	3,00
206	,00	,00	1,00	1,00
207	,00	,00	1,00	1,00
208	1,00	1,00	2,00	3,00
209	,00	,00	1,00	1,00
210	1,00	1,00	2,00	2,00
211	,00	,00	1,00	1,00
212	,00	,00	1,00	3,00
213	,00	,00	2,00	2,00
214	,00	,00	2,00	1,00
215	,00	,00	2,00	1,00
216	,00	,00	2,00	1,00
217	1,00	,00	2,00	3,00
218	,00	,00	1,00	1,00
219	,00	,00	1,00	1,00
220	1,00	,00	2,00	3,00
221	,00	,00	2,00	1,00
222	1,00	,00	2,00	3,00

	Morphology_coded	Grading_Paktuell April2013	ECOG	Prop_PD1
186	1,00	2	0	,00
187	1,00	1	0	,00
188	1,00	2	0	,00
189	1,00	1	0	,00
190	1,00	2	1	,00
191	1,00	3	0	,00
192	1,00	2	2	,00
193	1,00	1	0	,00
194	1,00	1	0	,00
195	1,00	2	0	,00
196	1,00	3	0	,00
197	1,00	3	0	,00
198	1,00	2	0	,00
199	1,00	1	0	,00
200	1,00	1	0	,00
201	1,00	3	.	,00
202	1,00	2	0	,00
203	1,00	3	0	,00
204	1,00	2	0	,00
205	1,00	2	0	,00
206	1,00	2	0	,00
207	1,00	2	0	,00
208	1,00	3	0	,00
209	1,00	2	0	,00
210	1,00	3	0	,00
211	1,00	1	0	,00
212	1,00	2	0	,00
213	1,00	3	0	1,00
214	1,00	1	0	,00
215	1,00	1	0	,00
216	1,00	2	0	,00
217	1,00	2	0	,00
218	1,00	1	0	,00
219	1,00	1	0	,00
220	1,00	3	3	,00
221	1,00	2	1	,00
222	1,00	2	1	,00

	Intens_PD1	PD1IRS	Prop_PDL1_TU	Intens_PDL1_TU	PDL1_TU_IRS
186	.	,00	,00	.	,00
187	.	,00	,00	.	,00
188	.	,00	,00	.	,00
189	.	,00	,00	.	,00
190	.	,00	,00	.	,00
191	.	,00	,00	.	,00
192	.	,00	,00	.	,00
193	.	,00	,00	.	,00
194	.	,00	,00	.	,00
195	.	,00	,00	.	,00
196	.	,00	,00	.	,00
197	.	,00	,00	.	,00
198	.	,00	,00	.	,00
199	.	,00	,00	.	,00
200	.	,00	,00	.	,00
201	.	,00	,00	.	,00
202	.	,00	,00	.	,00
203	.	,00	,00	.	,00
204	.	,00	,00	.	,00
205	.	,00	,00	.	,00
206	.	,00	,00	.	,00
207	.	,00	,00	.	,00
208	.	,00	,00	.	,00
209	.	,00	,00	.	,00
210	.	,00	,00	.	,00
211	.	,00	,00	.	,00
212	.	,00	,00	.	,00
213	1,00	1,00	3,00	2,00	6,00
214	.	,00	,00	.	,00
215	.	,00	,00	.	,00
216	.	,00	,00	.	,00
217	.	,00	,00	.	,00
218	.	,00	,00	.	,00
219	.	,00	,00	.	,00
220	.	,00	,00	.	,00
221	.	,00	,00	.	,00
222	.	,00	,00	.	,00

RCC for Publication.sav

	Prop_PDL1_Lymph	Intens_PDL1_Lymph	PDL1_Lymph_IRS	andreasProp_Lymph_CTLA4_TU
186	.	.	,00	,00
187	.	.	,00	,00
188	.	.	,00	,00
189	.	.	,00	,00
190	.	.	,00	,00
191	.	.	,00	,00
192	.	.	,00	,00
193	.	.	,00	,00
194	.	.	,00	,00
195	.	.	,00	,00
196	.	.	,00	,00
197	.	.	,00	,00
198	.	.	,00	,00
199	.	.	,00	,00
200	.	.	,00	,00
201	.	.	,00	,00
202	.	.	,00	,00
203	.	.	,00	,00
204	.	.	,00	,00
205	.	.	,00	,00
206	.	.	,00	,00
207	.	.	,00	,00
208	.	.	,00	,00
209	.	.	,00	,00
210	.	.	,00	,00
211	.	.	,00	,00
212	.	.	,00	,00
213	,00	,00	,00	,00
214	.	.	,00	,00
215	.	.	,00	,00
216	.	.	,00	,00
217	.	.	,00	,00
218	.	.	,00	,00
219	.	.	,00	,00
220	.	.	,00	,00
221	.	.	,00	,00
222	.	.	,00	,00

	CD3medianbis2k5unddarüber
186	1,00
187	,00
188	,00
189	1,00
190	1,00
191	,00
192	,00
193	1,00
194	,00
195	,00
196	1,00
197	1,00
198	,00
199	1,00
200	1,00
201	,00
202	,00
203	1,00
204	,00
205	1,00
206	,00
207	,00
208	,00
209	,00
210	1,00
211	,00
212	1,00
213	1,00
214	1,00
215	,00
216	,00
217	,00
218	1,00
219	,00
220	,00
221	,00
222	1,00

	Diagnosedatum_Primär	Met_EarliestDatum	Last_contact_Datum	AgeAtDiagnosis	Diagnosis_to_lastcontact
223	03.11.2008	03.11.2008	15.03.2009	84	4
224	15.11.2008	.	01.07.2012	39	43
225	18.12.2008	.	25.10.2009	72	10
226	15.02.2009	.	01.06.2012	47	39
227	16.02.2009	05.09.2011	14.11.2011	59	32
228	18.03.2009	.	09.02.2012	56	34
229	20.03.2009	.	01.08.2012	71	40
230	21.04.2009	.	01.02.2012	39	33
231	29.04.2009	29.12.2009	01.07.2012	74	38
232	02.05.2009	.	21.05.2012	73	36
233	20.05.2009	.	23.05.2012	76	36
234	22.06.2009	.	21.05.2012	60	34
235	02.07.2009	.	21.05.2012	55	34
236	15.07.2009	29.08.2010	29.08.2010	85	13
237	22.07.2009	.	01.08.2012	69	36
238	01.08.2009	.	01.07.2012	66	35
239	06.10.2009	.	01.08.2012	79	33
240	15.10.2009	.	01.07.2012	75	32
241	02.11.2009	.	21.05.2012	55	30
242	02.11.2009	26.04.2011	01.07.2012	68	31
243	20.11.2009	.	01.08.2012	68	32
244	01.12.2009	.	01.07.2012	79	31
245	07.12.2009	29.03.2012	01.06.2012	61	29
246	15.12.2009	.	10.02.2012	77	25
247	15.12.2009	15.12.2009	10.02.2012	81	25
248	15.12.2009	15.12.2009	28.06.2012	52	30
249	15.01.2010	15.01.2010	21.05.2012	70	28
250	15.01.2010	15.01.2010	14.06.2012	54	28
251	15.01.2010	.	01.06.2012	47	28
252	08.02.2010	.	10.02.2012	77	24
253	15.02.2010	.	16.05.2012	75	27
254	15.02.2010	.	10.02.2012	47	23
255	02.03.2010	.	17.06.2010	50	3
256	02.03.2010	.	10.02.2012	50	23
257	15.03.2010	21.06.2010	02.03.2012	57	23
258	15.03.2010	.	10.02.2012	42	22
259	15.03.2010	.	21.05.2012	73	26

	Survival_OS	Survival_DSS	Geschlecht	pT_grouped
223	1,00	1,00	2,00	3,00
224	,00	,00	2,00	1,00
225	1,00	,00	2,00	3,00
226	,00	,00	2,00	1,00
227	1,00	,00	1,00	1,00
228	,00	,00	2,00	3,00
229	,00	,00	2,00	1,00
230	,00	,00	2,00	1,00
231	,00	,00	1,00	1,00
232	,00	,00	1,00	1,00
233	,00	,00	2,00	1,00
234	,00	,00	2,00	1,00
235	,00	,00	1,00	1,00
236	1,00	,00	2,00	1,00
237	,00	,00	2,00	1,00
238	,00	,00	2,00	1,00
239	,00	,00	1,00	1,00
240	,00	,00	1,00	3,00
241	,00	,00	2,00	3,00
242	,00	,00	1,00	1,00
243	,00	,00	2,00	2,00
244	,00	,00	2,00	1,00
245	,00	,00	2,00	1,00
246	,00	,00	2,00	1,00
247	,00	,00	1,00	1,00
248	,00	,00	2,00	3,00
249	,00	,00	2,00	3,00
250	,00	,00	1,00	3,00
251	,00	,00	2,00	2,00
252	,00	,00	1,00	3,00
253	,00	,00	2,00	1,00
254	,00	,00	2,00	1,00
255	,00	,00	2,00	1,00
256	,00	,00	1,00	1,00
257	,00	,00	1,00	1,00
258	,00	,00	2,00	1,00
259	,00	,00	2,00	1,00

	Morphology_coded	Grading_Paktuell April2013	ECOG	Prop_PD1
223	1,00	3	1	,00
224	1,00	2	0	,00
225	1,00	2	1	,00
226	1,00	3	0	,00
227	1,00	1	0	,00
228	1,00	2	0	,00
229	1,00	2	0	,00
230	1,00	2	.	,00
231	1,00	1	0	,00
232	1,00	2	1	,00
233	1,00	2	0	,00
234	1,00	2	0	,00
235	1,00	2	0	,00
236	1,00	2	0	,00
237	1,00	2	0	,00
238	1,00	2	0	,00
239	1,00	1	1	,00
240	1,00	3	.	,00
241	1,00	3	2	,00
242	1,00	2	0	,00
243	1,00	3	0	,00
244	1,00	1	1	,00
245	1,00	3	0	,00
246	1,00	2	0	,00
247	1,00	1	0	,00
248	1,00	2	0	,00
249	1,00	2	1	,00
250	1,00	3	0	,00
251	1,00	2	0	,00
252	1,00	3	1	,00
253	1,00	2	1	,00
254	1,00	2	1	,00
255	1,00	2	0	,00
256	1,00	2	0	,00
257	1,00	2	0	.
258	1,00	2	0	,00
259	1,00	2	0	,00

	Intens_PD1	PD1IRS	Prop_PDL1_TU	Intens_PDL1_TU	PDL1_TU_IRS
223	.	,00	,00	.	,00
224	.	,00	,00	.	,00
225	.	,00	,00	.	,00
226	.	,00	,00	.	,00
227	.	,00	,00	.	,00
228	.	,00	,00	.	,00
229	.	,00	,00	.	,00
230	.	,00	,00	.	,00
231	.	,00	,00	.	,00
232	.	,00	,00	.	,00
233	.	,00	,00	.	,00
234	.	,00	,00	.	,00
235	.	,00	,00	.	,00
236	.	,00	,00	.	,00
237	.	,00	,00	.	,00
238	.	,00	,00	.	,00
239	.	,00	,00	.	,00
240	.	,00	,00	.	,00
241	.	,00	,00	.	,00
242	.	,00	,00	.	,00
243	.	,00	,00	.	,00
244	.	,00	,00	.	,00
245	.	,00	,00	.	,00
246	.	,00	,00	.	,00
247	.	,00	,00	.	,00
248	.	,00	,00	.	,00
249	.	,00	,00	.	,00
250	.	,00	,00	.	,00
251	.	,00	,00	.	,00
252	.	,00	,00	.	,00
253	.	,00	,00	.	,00
254	.	,00	,00	.	,00
255	.	,00	,00	.	,00
256	.	,00	,00	.	,00
257	.	.	,00	,00	,00
258	.	,00	,00	.	,00
259	.	,00	,00	.	,00

RCC for Publication.sav

	Prop_PDL1_Lymph	Intens_PDL1_Lymph	PDL1_Lymph_IRS	andreasProp_Lymph_CTLA4_TU
223	.	.	,00	,00
224	.	.	,00	,00
225	.	.	,00	1,00
226	.	.	,00	,00
227	.	.	,00	,00
228	.	.	,00	,00
229	.	.	,00	,00
230	.	.	,00	2,00
231	.	.	,00	,00
232	.	.	,00	,00
233	.	.	,00	,00
234	.	.	,00	,00
235	.	.	,00	,00
236	.	.	,00	,00
237	.	.	,00	,00
238	.	.	,00	,00
239	.	.	,00	,00
240	.	.	,00	,00
241	.	.	,00	,00
242	.	.	,00	,00
243	.	.	,00	,00
244	.	.	,00	2,00
245	.	.	,00	,00
246	.	.	,00	,00
247	.	.	,00	,00
248	.	.	,00	1,00
249	.	.	,00	,00
250	.	.	,00	,00
251	.	.	,00	,00
252	.	.	,00	,00
253	.	.	,00	,00
254	.	.	,00	,00
255	.	.	,00	,00
256	.	.	,00	,00
257	,00	,00	,00	,00
258	.	.	,00	,00
259	.	.	,00	,00

	CD3medianbis2k5unddarüber
223	1,00
224	,00
225	1,00
226	1,00
227	,00
228	,00
229	1,00
230	1,00
231	1,00
232	1,00
233	1,00
234	,00
235	1,00
236	1,00
237	1,00
238	1,00
239	,00
240	1,00
241	1,00
242	,00
243	1,00
244	,00
245	1,00
246	1,00
247	,00
248	,00
249	1,00
250	1,00
251	.
252	1,00
253	1,00
254	1,00
255	1,00
256	,00
257	,00
258	,00
259	,00

RCC for Publication.sav

	Diagnosedatum_Primär	Met_EarliestDatum	Last_contact_Datum	AgeAtDiagnosis	Diagnosis_to_lastcontact
260	31.03.2010	.	18.06.2012	23	26
261	15.04.2010	.	21.05.2012	62	25
262	02.05.2010	.	01.07.2012	59	25
263	15.05.2010	15.05.2010	08.06.2012	71	24
264	25.05.2010	.	03.04.2012	61	22
265	07.06.2010	.	29.05.2012	53	23
266	15.06.2010	.	03.04.2012	69	21
267	15.06.2010	.	21.05.2012	82	23
268	06.07.2010	.	11.07.2012	56	24
269	06.07.2010	.	01.07.2012	68	23
270	26.07.2010	.	02.04.2012	49	20
271	16.08.2010	.	01.06.2012	68	21
272	15.09.2010	.	21.05.2012	49	20
273	04.10.2010	.	23.05.2012	60	19
274	10.01.2011	.	26.03.2012	53	14
275	18.01.2011	.	21.05.2012	61	16
276	03.03.2011	.	27.03.2012	66	12
277	14.03.2011	.	24.02.2012	53	11
278	15.03.2011	.	01.07.2012	69	15
279	05.04.2011	.	15.03.2012	70	11
280	11.04.2011	.	01.08.2012	65	15
281	15.04.2011	.	01.07.2012	41	14
282	19.05.2011	.	23.05.2012	69	12
283	22.06.2011	.	11.06.2012	59	11
284	11.07.2011	.	01.03.2012	68	7
285	12.07.2011	.	23.03.2012	32	8
286	21.09.2011	.	24.05.2012	63	8
287	21.09.2011	.	19.07.2012	69	9
288	27.09.2011	.	01.07.2012	62	9
289	08.11.2011	.	25.03.2012	74	4
290	17.09.1998	.	07.02.2012	77	160
291	08.03.2005	08.03.2005	05.06.2005	68	2
292	04.12.2009	04.12.2009	14.06.2012	70	30
293	15.07.2010	.	01.06.2012	65	22
294	17.02.2005	.	18.05.2012	76	87
295	24.10.2011	.	01.07.2012	53	8
296	10.01.2002	.	01.11.2011	60	117

RCC for Publication.sav

	Survival_OS	Survival_DSS	Geschlecht	pT_grouped
260	,00	,00	2,00	1,00
261	,00	,00	2,00	1,00
262	,00	,00	2,00	1,00
263	,00	,00	1,00	1,00
264	,00	,00	2,00	1,00
265	,00	,00	2,00	.
266	,00	,00	1,00	1,00
267	,00	,00	2,00	3,00
268	,00	,00	1,00	3,00
269	,00	,00	2,00	1,00
270	,00	,00	2,00	1,00
271	,00	,00	2,00	1,00
272	,00	,00	2,00	1,00
273	,00	,00	1,00	1,00
274	,00	,00	2,00	1,00
275	,00	,00	1,00	1,00
276	,00	,00	2,00	1,00
277	,00	,00	1,00	1,00
278	,00	,00	2,00	1,00
279	,00	,00	2,00	1,00
280	,00	,00	2,00	1,00
281	,00	,00	2,00	2,00
282	,00	,00	2,00	1,00
283	,00	,00	2,00	1,00
284	,00	,00	2,00	2,00
285	,00	,00	1,00	1,00
286	,00	,00	1,00	3,00
287	,00	,00	2,00	2,00
288	,00	,00	1,00	1,00
289	,00	,00	1,00	1,00
290	,00	,00	2,00	1,00
291	1,00	,00	2,00	3,00
292	,00	,00	2,00	1,00
293	,00	,00	2,00	1,00
294	,00	,00	1,00	1,00
295	,00	,00	2,00	1,00
296	1,00	,00	2,00	1,00

RCC for Publication.sav

	Morphology_coded	Grading_Paktuell April2013	ECOG	Prop_PD1
260	1,00	1	0	,00
261	1,00	3	0	,00
262	1,00	1	1	,00
263	1,00	2	0	,00
264	1,00	2	0	,00
265	1,00	1	.	,00
266	1,00	2	0	,00
267	1,00	2	0	,00
268	1,00	2	0	,00
269	1,00	2	1	,00
270	1,00	2	0	,00
271	1,00	2	0	,00
272	1,00	2	.	,00
273	1,00	2	0	,00
274	1,00	1	0	,00
275	1,00	1	0	,00
276	1,00	2	0	,00
277	1,00	2	0	,00
278	1,00	2	0	,00
279	1,00	2	0	,00
280	1,00	1	.	,00
281	1,00	2	0	,00
282	1,00	2	.	,00
283	1,00	2	0	,00
284	1,00	1	.	,00
285	1,00	1	0	,00
286	1,00	2	0	,00
287	1,00	2	1	,00
288	1,00	2	.	,00
289	1,00	2	.	,00
290	1,00	2	.	,00
291	1,00	3	0	,00
292	1,00	2	1	,00
293	1,00	2	0	,00
294	1,00	3	0	,00
295	1,00	2	0	,00
296	1,00	2	.	,00

	Intens_PD1	PD1IRS	Prop_PDL1_TU	Intens_PDL1_TU	PDL1_TU_IRS
260	.	,00	,00	.	,00
261	.	,00	,00	.	,00
262	.	,00	,00	.	,00
263	.	,00	,00	.	,00
264	.	,00	,00	.	,00
265	.	,00	,00	.	,00
266	.	,00	,00	.	,00
267	.	,00	,00	.	,00
268	.	,00	,00	.	,00
269	.	,00	,00	.	,00
270	.	,00	,00	.	,00
271	.	,00	,00	.	,00
272	.	,00	,00	.	,00
273	.	,00	,00	.	,00
274	.	,00	,00	.	,00
275	.	,00	,00	.	,00
276	.	,00	,00	.	,00
277	.	,00	,00	.	,00
278	.	,00	,00	.	,00
279	.	,00	,00	.	,00
280	.	,00	,00	.	,00
281	.	,00	,00	.	,00
282	.	,00	,00	.	,00
283	.	,00	,00	.	,00
284	.	,00	,00	.	,00
285	.	,00	,00	.	,00
286	.	,00	,00	.	,00
287	.	,00	,00	.	,00
288	.	,00	,00	.	,00
289	.	,00	,00	.	,00
290	.	,00	,00	.	,00
291	.	,00	,00	.	,00
292	.	,00	,00	.	,00
293	.	,00	,00	.	,00
294	.	,00	2,00	1,00	2,00
295	.	,00	2,00	1,00	2,00
296	.	,00	2,00	3,00	2,00

	Prop_PDL1_Lymph	Intens_PDL1_Lymph	PDL1_Lymph_IRS	andreasProp_Lymph_CTLA4_TU
260	.	.	,00	,00
261	.	.	,00	,00
262	.	.	,00	,00
263	.	.	,00	,00
264	.	.	,00	,00
265	.	.	,00	,00
266	.	.	,00	,00
267	.	.	,00	,00
268	.	.	,00	,00
269	.	.	,00	,00
270	.	.	,00	,00
271	.	.	,00	,00
272	.	.	,00	,00
273	.	.	,00	,00
274	.	.	,00	,00
275	.	.	,00	,00
276	.	.	,00	,00
277	.	.	,00	,00
278	.	.	,00	,00
279	.	.	,00	,00
280	.	.	,00	,00
281	.	.	,00	,00
282	.	.	,00	,00
283	.	.	,00	,00
284	.	.	,00	,00
285	.	.	,00	,00
286	.	.	,00	,00
287	.	.	,00	,00
288	.	.	,00	,00
289	.	.	,00	,00
290	.	.	,00	10,00
291	.	.	,00	5,00
292	.	.	,00	10,00
293	.	.	,00	10,00
294	.	.	,00	.
295	.	.	,00	.
296	.	.	,00	,00

	CD3medianbis2k5unddarüber
260	,00
261	1,00
262	1,00
263	,00
264	,00
265	,00
266	,00
267	1,00
268	1,00
269	,00
270	,00
271	1,00
272	,00
273	1,00
274	,00
275	1,00
276	,00
277	,00
278	1,00
279	1,00
280	,00
281	.
282	1,00
283	1,00
284	,00
285	,00
286	1,00
287	,00
288	1,00
289	,00
290	,00
291	1,00
292	1,00
293	1,00
294	1,00
295	.
296	,00

	Diagnosedatum_Primär	Met_EarliestDatum	Last_contact_Datum	AgeAtDiagnosis	Diagnosis_to_lastcontact
297	22.12.2003	.	01.07.2012	64	102
298	25.05.2004	.	22.03.2010	61	69
299	28.07.2004	.	11.08.2006	67	24
300	29.04.2005	.	01.08.2012	28	87
301	02.08.2005	02.08.2005	21.04.2011	77	68
302	25.01.2006	.	01.07.2012	67	77
303	31.03.2006	.	01.08.2012	60	76
304	11.05.2006	.	01.07.2012	54	73
305	15.03.2007	15.03.2007	15.06.2008	72	15
306	08.05.2008	.	01.06.2012	83	48
307	11.06.2010	.	11.04.2012	51	22
308	06.03.2008	16.07.2008	18.07.2008	66	4
309	19.07.2011	.	01.08.2012	63	12
310	02.07.2007	.	19.03.2012	60	56
311	16.03.1999	29.03.2010	01.07.2012	50	159
312	15.04.2006	.	01.07.2012	67	74
313	24.04.2007	.	16.05.2012	61	60
314	09.06.2008	.	01.07.2012	68	48
315	10.09.2008	.	02.01.2009	72	3
316	08.10.2009	.	10.02.2012	67	28
317	09.06.2006	.	01.07.2012	46	72
318	29.07.2009	15.02.2011	19.05.2011	84	21
319	15.10.2009	.	10.02.2012	77	27
320	15.04.2010	.	21.05.2012	69	25
321	06.09.2005	.	01.07.2012	44	81
322	29.09.2005	29.09.2005	13.07.2006	52	9
323	18.02.2009	.	01.07.2012	57	40
324	24.03.2009	02.10.2009	13.08.2010	72	16
325	03.07.2009	20.07.2010	22.05.2012	54	34
326	02.09.2009	02.09.2009	21.05.2012	59	32
327	01.07.2010	01.07.2010	19.09.2010	76	2
328	30.08.2010	.	21.05.2012	61	20
329	01.10.2010	.	21.05.2012	56	19
330	15.01.2011	15.05.2011	01.06.2012	60	16
331	19.04.2003	29.07.2003	13.08.2003	76	3
332	02.08.2007	02.08.2007	14.06.2012	73	58
333	25.05.2009	25.05.2009	24.08.2011	74	26

	Survival_OS	Survival_DSS	Geschlecht	pT_grouped
297	,00	,00	2,00	1,00
298	1,00	,00	2,00	3,00
299	1,00	,00	1,00	1,00
300	,00	,00	2,00	3,00
301	1,00	,00	2,00	3,00
302	,00	,00	2,00	3,00
303	,00	,00	2,00	1,00
304	,00	,00	2,00	1,00
305	1,00	1,00	1,00	3,00
306	,00	,00	1,00	3,00
307	,00	,00	2,00	1,00
308	1,00	1,00	2,00	3,00
309	,00	,00	2,00	1,00
310	,00	,00	1,00	1,00
311	,00	,00	1,00	3,00
312	,00	,00	1,00	1,00
313	,00	,00	1,00	1,00
314	,00	,00	1,00	1,00
315	1,00	,00	2,00	3,00
316	,00	,00	1,00	1,00
317	,00	,00	2,00	1,00
318	1,00	,00	2,00	1,00
319	1,00	,00	2,00	3,00
320	,00	,00	1,00	1,00
321	,00	,00	2,00	1,00
322	1,00	1,00	2,00	3,00
323	,00	,00	2,00	2,00
324	1,00	,00	2,00	1,00
325	,00	,00	2,00	3,00
326	,00	,00	2,00	3,00
327	1,00	,00	2,00	3,00
328	,00	,00	2,00	2,00
329	,00	,00	1,00	1,00
330	,00	,00	2,00	2,00
331	1,00	1,00	1,00	3,00
332	,00	,00	1,00	4,00
333	1,00	,00	2,00	3,00

RCC for Publication.sav

	Morphology_coded	Grading_Paktuell April2013	ECOG	Prop_PD1
297	1,00	3	0	,00
298	1,00	3	0	,00
299	1,00	1	0	,00
300	1,00	2	0	,00
301	1,00	3	1	,00
302	1,00	3	0	,00
303	1,00	3	0	,00
304	1,00	2	0	,00
305	1,00	3	1	,00
306	1,00	2	1	,00
307	1,00	2	1	,00
308	1,00	3	0	,00
309	1,00	2	0	,00
310	1,00	3	.	,00
311	1,00	2	0	,00
312	1,00	2	1	,00
313	1,00	3	0	,00
314	1,00	2	0	,00
315	1,00	2	0	,00
316	1,00	1	0	,00
317	1,00	2	0	,00
318	1,00	3	.	,00
319	1,00	3	0	1,00
320	1,00	2	1	1,00
321	1,00	2	1	1,00
322	1,00	3	0	1,00
323	1,00	3	0	1,00
324	1,00	3	2	1,00
325	1,00	3	0	1,00
326	1,00	2	0	1,00
327	1,00	3	2	2,00
328	1,00	2	0	1,00
329	1,00	2	0	1,00
330	1,00	3	.	1,00
331	1,00	3	.	1,00
332	1,00	2	.	1,00
333	1,00	2	1	1,00

	Intens_PD1	PD1IRS	Prop_PDL1_TU	Intens_PDL1_TU	PDL1_TU_IRS
297	.	,00	2,00	1,00	2,00
298	.	,00	1,00	2,00	1,00
299	.	,00	3,00	1,00	3,00
300	.	,00	1,00	2,00	2,00
301	.	,00	2,00	1,00	2,00
302	.	,00	1,00	1,00	1,00
303	.	,00	1,00	1,00	1,00
304	.	,00	1,00	1,00	1,00
305	.	,00	2,00	1,00	2,00
306	.	,00	2,00	3,00	6,00
307	.	,00	1,00	1,00	1,00
308	.	,00	2,00	1,00	2,00
309	.	,00	2,00	1,00	2,00
310	.	,00	,00	.	,00
311	.	,00	,00	.	,00
312	.	,00	,00	.	,00
313	.	,00	,00	.	,00
314	.	,00	,00	.	,00
315	.	,00	,00	.	,00
316	.	,00	,00	.	,00
317	.	,00	2,00	2,00	4,00
318	,00	,00	,00	,00	,00
319	1,00	1,00	,00	.	,00
320	2,00	2,00	,00	.	,00
321	1,00	1,00	,00	.	,00
322	1,00	1,00	,00	.	,00
323	1,00	1,00	,00	.	,00
324	1,00	1,00	,00	.	,00
325	1,00	1,00	,00	.	,00
326	1,00	1,00	,00	.	,00
327	3,00	6,00	,00	.	,00
328	1,00	1,00	,00	.	,00
329	1,00	1,00	,00	.	,00
330	1,00	1,00	,00	.	,00
331	1,00	1,00	,00	.	,00
332	1,00	1,00	,00	.	,00
333	1,00	1,00	,00	.	,00

	Prop_PDL1_Lymph	Intens_PDL1_Lymph	PDL1_Lymph_IRS	andreasProp_Lymph_CTLA4_TU
297	.	.	,00	2,00
298	.	.	,00	1,00
299	.	.	,00	,00
300	.	.	,00	,00
301	.	.	,00	,00
302	.	.	,00	,00
303	.	.	,00	,00
304	.	.	,00	,00
305	.	.	,00	,00
306	.	.	,00	,00
307	.	.	,00	,00
308	.	.	,00	10,00
309	.	.	,00	5,00
310	2,00	1,00	2,00	.
311	2,00	2,00	4,00	1,00
312	1,00	1,00	1,00	1,00
313	1,00	1,00	1,00	1,00
314	2,00	1,00	2,00	,00
315	1,00	1,00	1,00	,00
316	1,00	2,00	2,00	,00
317	1,00	1,00	1,00	10,00
318	,00	,00	,00	.
319	.	.	,00	.
320	.	.	,00	.
321	.	.	,00	1,00
322	.	.	,00	,00
323	.	.	,00	2,00
324	.	.	,00	,00
325	.	.	,00	,00
326	.	.	,00	,00
327	.	.	,00	,00
328	.	.	,00	,00
329	.	.	,00	,00
330	.	.	,00	,00
331	.	.	,00	10,00
332	.	.	,00	5,00
333	.	.	,00	20,00

	CD3medianbis2k5unddarüber
297	,00
298	,00
299	,00
300	1,00
301	1,00
302	1,00
303	1,00
304	,00
305	,00
306	1,00
307	.
308	1,00
309	1,00
310	,00
311	1,00
312	1,00
313	,00
314	,00
315	,00
316	,00
317	1,00
318	1,00
319	1,00
320	1,00
321	1,00
322	1,00
323	1,00
324	1,00
325	1,00
326	1,00
327	1,00
328	1,00
329	1,00
330	1,00
331	1,00
332	1,00
333	1,00

	Diagnosedatum_Primär	Met_EarliestDatum	Last_contact_Datum	AgeAtDiagnosis	Diagnosis_to_lastcontact
334	03.02.2010	.	05.03.2010	79	1
335	11.03.2011	.	01.07.2012	40	15
336	12.01.2004	.	01.07.2012	74	101
337	14.07.2005	.	06.05.2011	73	69
338	20.09.2011	.	04.04.2012	55	6
339	09.05.2009	09.05.2009	06.08.2009	71	2
340	15.02.2010	.	10.02.2012	78	23
341	14.07.2011	.	05.04.2012	57	8
342	03.03.2008	.	01.07.2012	70	51
343					
344					
345					
346					
347					
348					
349					
350					
351					
352					
353					
354					
355					
356					
357					
358					
359					
360					
361					
362					
363					
364					
365					
366					
367					
368					
369					
370					

RCC for Publication.sav

	Survival_OS	Survival_DSS	Geschlecht	pT_grouped
334	1,00	1,00	2,00	3,00
335	,00	,00	2,00	1,00
336	,00	,00	2,00	1,00
337	1,00	,00	2,00	1,00
338	,00	,00	2,00	1,00
339	1,00	1,00	1,00	3,00
340	,00	,00	2,00	1,00
341	,00	,00	1,00	1,00
342	,00	,00	1,00	1,00
343				
344				
345				
346				
347				
348				
349				
350				
351				
352				
353				
354				
355				
356				
357				
358				
359				
360				
361				
362				
363				
364				
365				
366				
367				
368				
369				
370				

	Morphology_coded	Grading_Paktuell April2013	ECOG	Prop_PD1
334	1,00	2	1	1,00
335	1,00	2	0	1,00
336	1,00	3	0	2,00
337	1,00	2	0	1,00
338	1,00	3	0	1,00
339	1,00	3	1	1,00
340	1,00	2	0	1,00
341	1,00	1	0	,00
342	1,00	2	0	1,00
343				
344				
345				
346				
347				
348				
349				
350				
351				
352				
353				
354				
355				
356				
357				
358				
359				
360				
361				
362				
363				
364				
365				
366				
367				
368				
369				
370				

	Intens_PD1	PD1IRS	Prop_PDL1_TU	Intens_PDL1_TU	PDL1_TU_IRS
334	1,00	1,00	,00	.	,00
335	1,00	1,00	2,00	1,00	2,00
336	1,00	2,00	1,00	1,00	1,00
337	1,00	1,00	1,00	1,00	1,00
338	2,00	2,00	2,00	1,00	2,00
339	2,00	2,00	1,00	2,00	2,00
340	1,00	1,00	2,00	2,00	4,00
341	.	,00	3,00	2,00	6,00
342	1,00	1,00	,00	.	,00
343					
344					
345					
346					
347					
348					
349					
350					
351					
352					
353					
354					
355					
356					
357					
358					
359					
360					
361					
362					
363					
364					
365					
366					
367					
368					
369					
370					

	Prop_PDL1_Lymph	Intens_PDL1_Lymph	PDL1_Lymph_IRS	andreasProp_Lymph_CTLA4_TU
334	.	.	,00	5,00
335	.	.	,00	.
336	.	.	,00	1,00
337	.	.	,00	,00
338	.	.	,00	,00
339	.	.	,00	5,00
340	.	.	,00	10,00
341	.	.	,00	5,00
342	2,00	2,00	4,00	2,00
343				
344				
345				
346				
347				
348				
349				
350				
351				
352				
353				
354				
355				
356				
357				
358				
359				
360				
361				
362				
363				
364				
365				
366				
367				
368				
369				
370				

	CD3medianbis2k5unddarüber
334	1,00
335	1,00
336	1,00
337	1,00
338	1,00
339	1,00
340	1,00
341	1,00
342	,00
343	
344	
345	
346	
347	
348	
349	
350	
351	
352	
353	
354	
355	
356	
357	
358	
359	
360	
361	
362	
363	
364	
365	
366	
367	
368	
369	
370	

RCC for Publication.sav

	Diagnosedatum_Primär	Met_EarliestDatum	Last_contact_Datum	AgeAtDiagnosis	Diagnosis_to_lastcontact
371					
372					
373					
374					
375					
376					
377					
378					
379					
380					
381					
382					

RCC for Publication.sav

	Survival_OS	Survival_DSS	Geschlecht	pT_grouped
371				
372				
373				
374				
375				
376				
377				
378				
379				
380				
381				
382				

RCC for Publication.sav

	Morphology_coded	Grading_Paktuell April2013	ECOG	Prop_PD1
371				
372				
373				
374				
375				
376				
377				
378				
379				
380				
381				
382				

	Intens_PD1	PD1IRS	Prop_PDL1_TU	Intens_PDL1_TU	PDL1_TU_IRS
371					
372					
373					
374					
375					
376					
377					
378					
379					
380					
381					
382					

RCC for Publication.sav

	Prop_PDL1_Lymph	Intens_PDL1_Lymph	PDL1_Lymph_IRS	andreasProp_Lymph_CTLA4_TU
371				
372				
373				
374				
375				
376				
377				
378				
379				
380				
381				
382				

	CD3medianbis2k5unddarüber
371	
372	
373	
374	
375	
376	
377	
378	
379	
380	
381	
382	
