# Supplementary material for: Expression of PD-1 and CTLA-4 Are Negative Prognostic Markers in Renal Cell Carcinoma
Source: J Clin Med. 2019 May 24;8(5):743. doi: 10.3390/jcm8050743 (PMC6572544; doi:10.3390/jcm8050743)
Supplement: Supplementary file 1 [file jcm-08-00743-s001.zip › jcm-502061 supplementary 2.pdf]

Supplementary Tables.

Table S1. Univariate Cox regression analysis: Association of clinical parameters and immunological markers with OS or CSS in all RCC patients.

All RCC	OS Hazard Ratio	Range		p-value	CSS Hazard Ratio	Range		p-value
PD-1 TIMC	2·466	1·362	4·465	0·003*	2·344	0·901	6·101	0·081
PD-L1 TU	1·130	0·614	2078	0·695	0·977	0·344	2·775	0·964
PD-L1 TIMC	0·793	0·290	2·165	0·650	No cancer specific death			
CTLA-4	2·442	1·176	5·067	0·017*	3·271	1·146	9·337	0·027*
PD-1 + CTLA-4	4·284	1·718	10·680	0·002**	6·317	1·898	21·026	0·003**
CD3	0·903	0·595	1·369	0·630	1·344	0·683	2·644	0·393
Age >65years	2·696	1·733	4·194	<0·001**	2·255	1·120	4·537	0·023*
Gender male	1·802	1·115	2·911	0·016*	1·278	0·626	2·610	0·646
Stage >pT2	3·352	2·216	5·071	<0·001**	3·029	1·544	5·943	0·001**
Grade =G3	3·546	2·353	5·346	<0·001**	6·661	3·354	13·231	<0·001**
ECOG >0	2·338	1·485	3·682	<0·001**	1·947	0·921	4·157	0·085

** Correlation is significant at 0·01 level (2-tailed) * Correlation is significant at 0·05 level (2-tailed).

Table S2. Univariate Cox regression analysis: Association of clinical parameters and immunological markers with OS or CSS in the no primary metastases subgroup.

No primary metastases	OS Hazard Ratio	Range		p-value	CSS Hazard Ratio	Range		p-value
PD-1 TIMC	2·108	0·956	4·647	0·065	2·053	0·469	8·980	0·339
PD-L1 TU	1·128	5·557	2·284	0·738	0·780	0·181	3·366	0·739
PD-L1 TIMC	1·000	0·363	2·758	1·000	No cancer specific death			
CTLA-4	2·516	1·004	6·300	0·049*	5·903	1·711	20·369	0·005**
PD-1 + CTLA-4	6·420	1·972	20·904	0·002**	16·349	3·526	75·818	<0·001**
CD3	0·702	0·425	1·160	0·168	0·988	0·401	2·431	0·979
Age >65years	2·374	1·438	3·919	0·001**	1·919	0·783	4·701	0·154
Gender male	1·824	1·039	3·203	0·036*	1·394	0·535	3·629	0·496
Stage >pT2	3·285	2·013	5·360	<0·001**	2·500	0·984	6·352	0·054
Grade =G3	2·514	1·524	4·145	<0·001**	4·417	1·838	10·615	0·001**
ECOG >0	2·187	1·256	3·807	0·006**	1·445	0·445	4·693	0·540

** Correlation is significant at 0·01 level (2-tailed) * Correlation is significant at 0·05 level (2-tailed).
